# Alpha-1 antitrypsin reduces inflammation and vasculopathy in mice with oxygen-induced retinopathy

**DOI:** 10.1186/s12950-025-00431-3

**Published:** 2025-02-11

**Authors:** Varaporn Suphapimol, Yu-Han Liu, Sandro Prato, Alexander Karnowski, Charles Hardy, Adriana Baz Morelli, Abhirup Jayasimhan, Devy Deliyanti, Jennifer L. Wilkinson-Berka

**Affiliations:** 1 Department of Anatomy and Physiology, School of Biomedical Sciences, The University of Melbourne, Parkville, VIC Australia; 2 CSL Limited, Melbourne, VIC Australia; 3 School of Biomedical Sciences, The University of Melbourne, Level 2, Medical Building 181, Grattan Street, Parkville, VIC 3010 Australia

**Keywords:** Alpha-1 antitrypsin, Oxygen-induced retinopathy, Inflammation, Microglia, Neovascularisation, Vascular leakage

## Abstract

**Background:**

Damage to the retinal vasculature is a major cause of vision loss and is influenced by a pro-inflammatory environment within retinal tissue. Alpha-1 antitrypsin (AAT) is a potent inhibitor of serine proteases and has anti-inflammatory properties. We hypothesised that AAT could reduce inflammation and vasculopathy in neovascular retinopathies including oxygen-induced retinopathy (OIR).

**Methods:**

Litters of C57BL/6J mice were randomised to develop OIR by exposure to high oxygen between postnatal days 7 to 12 resulting in vaso-obliteration (phase I OIR), and then room air from postnatal days 12 to 18 resulting in neovascularisation (phase II OIR). Control mice were exposed to room air. Separate cohorts of mice were administered control vehicle or human AAT (120 mg/kg) by intraperitoneal injection every second day in phase I or phase II OIR.

**Results:**

In phase I OIR, plasma levels of AAT were reduced compared to room air controls, and AAT treatment reduced vaso-obliteration. In phase II OIR, AAT treatment influenced inflammation by reducing the density of ionised calcium binding adaptor protein 1 + cells (microglia/macrophages) and modulating their cell process length and reducing mRNA levels of tumour necrosis factor and monocyte chemoattractant protein-1, but not interleukin-1b and interleukin-6 in retina. Furthermore, AAT treatment reduced retinal neovascularisation, gliosis, vascular endothelial growth factor mRNA and protein expression, and vascular leakage, compared to OIR controls.

**Conclusions:**

This research demonstrates the vasculo-protective actions of AAT, and thereby the potential of AAT as a therapeutic option for neovascular retinopathies.

**Supplementary Information:**

The online version contains supplementary material available at 10.1186/s12950-025-00431-3.

## Background

Neovascular retinopathies such as retinopathy of prematurity and proliferative diabetic retinopathy, are leading causes of vision loss and blindness in premature babies and the working population, respectively [1, 2]. They feature retinal neovascularisation and vascular permeability due to breakdown of the blood-retinal barrier, with both events involving the increased expression of vascular endothelial growth factor A (VEGFA) [3–5]. Indeed, the ocular delivery of anti-VEGF agents is a predominant treatment approach for proliferative diabetic retinopathy [5, 6] and emerging for children with retinopathy of prematurity [7]. Yet, anti-VEGF agents are not entirely protective in all patients with diabetic retinopathy [5] and there are safety concerns for retinopathy of prematurity including reduced circulating VEGFA levels leading to neurocognitive decline and pulmonary hypertension [8, 9]. This has led to the necessity to further understand the biological mechanisms underpinning the pathogenesis of neovascular retinopathies. An important mechanism is inflammation that contributes to retinal vascular disease through cytokines, chemokines, and other factors produced by certain immune cells [10–12]. These immune cell populations include microglia, macrophages, and CD8 + T cells that infiltrate the retina through a damaged blood-retinal barrier [10–12].

Alpha-1 antitrypsin (AAT) is a sialoglycoprotein encoded by the SERPINA1 gene that is mainly produced by liver hepatocytes and to a lesser extent pulmonary alveolar cells, tissue macrophages, blood monocytes, and neutrophils [13]. AAT is an inhibitor of serine proteases such as elastase, trypsin, thrombin, proteinase 3, and cathepsin G and acts to protect tissues from proteolytic tissue damage [13, 14]. Indeed, inherited genetic deficiency of AAT can result in liver disease and emphysema due to the loss of inhibition of serine proteases [15] with augmentation of AAT a recognised treatment approach [16]. In addition, AAT has immunomodulatory and anti-inflammatory properties through inhibition of serine proteases and possibly SERPIN activity independent mechanisms [17, 18]. Functioning as an acute phase protein, AAT released into the circulation can reach plasma levels three- to five-fold higher than normal in response to acute inflammation or infection. In addition to its anti-inflammatory properties, AAT has cytoprotective actions that are relevant to the retina [19–22].

In the current study we hypothesised that treatment with AAT would protect the vasculature in a murine model of retinopathy of prematurity known as oxygen-induced retinopathy (OIR).

## Methods

### Animals

All studies were approved by the University of Melbourne Animal Ethics Committee and adhered to the National Health and Medical Research Council of Australia’s Guidelines for the Care and Use of Animals in Scientific Research. Pregnant C57BL/6J mice (JAX stock number 000664) were obtained from the Animal Resources Centre (Perth, Western Australia). All mice were given a standard mouse diet (#SF00-100, Specialty Feeds, Perth, Australia), and housed at a consistent temperature of 21–22 °C under a 12-hour light/dark cycle.

### Oxygen-induced retinopathy

**Fig. 1:**
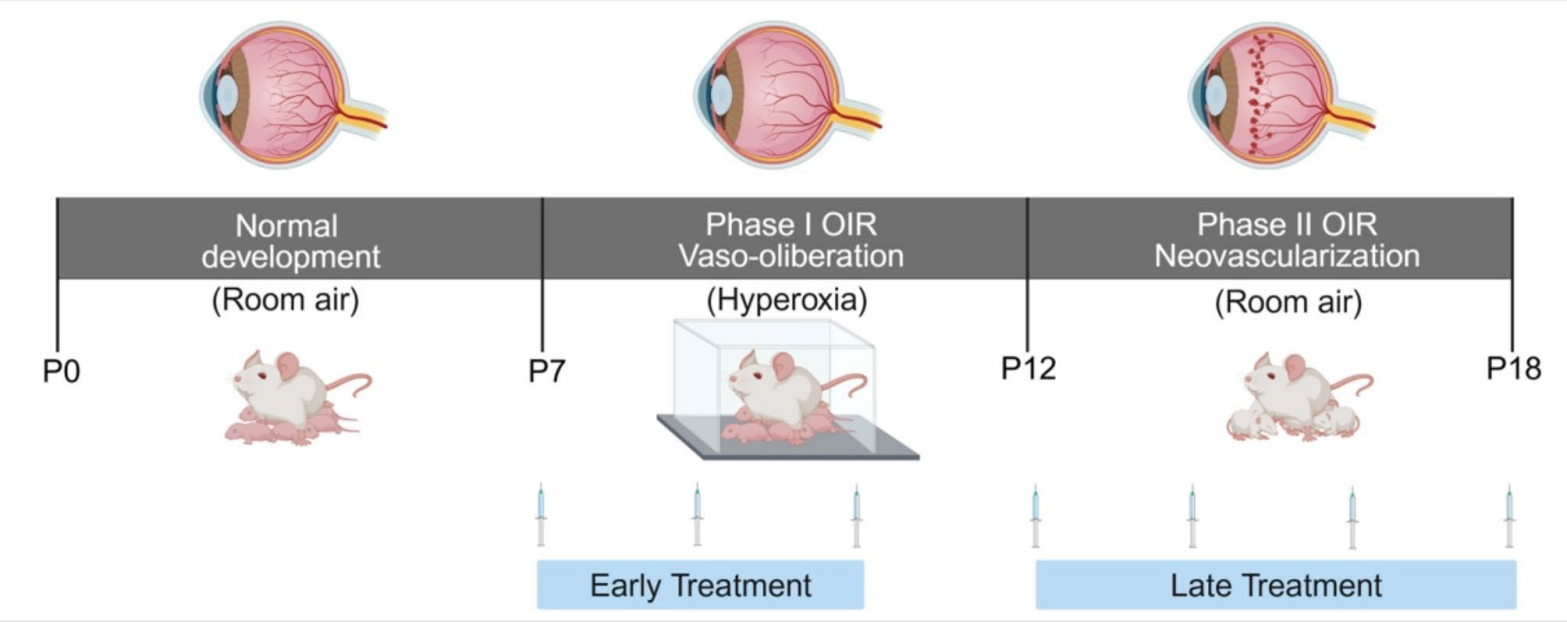
Schematic diagram showing the OIR protocol and administration of AAT. P, postnatal day. Separate groups of OIR mice were treated with AAT in phase I OIR (P7 to P12) or phase II OIR (P12 to P18). Created with BioRender.com

The OIR procedure occurs over two phases and was performed according to our previous publications [11, 23] and is a slightly modified version of the model established by Smith and colleagues [24]. Phase I OIR is characterized by extensive vaso-obliteration in the central retina due to the exposure of neonatal mice to hyperoxia from postnatal day (P) 7 to P12, that mimics when preterm infants receive supplemental oxygen to alleviate respiratory distress. Phase II OIR features neovascularization and vascular leakage due to the exposure of mice to room air for five days that induces retinal ischemia and the excessive production of vascular factors such as VEGFA [25]. In our study, litters of mice were randomised to control and OIR treatment groups. In phase I OIR, mouse pups and their nursing mothers were exposed to hyperoxia (75% oxygen) for 22 h per day between P7 to P12 in specialised chambers that were maintained by a Pro-ox 110 gas regulator (Biospherix, NY, USA) attached to medical-grade oxygen cylinders (Air Liquide, Victoria, Australia). Mice were then returned to room air until P18 (Fig. 1). Age-matched controls were housed in room air for the entirety of the study (21% oxygen).

To determine if AAT influenced the extent of vaso-obliteration that peaks in OIR during phase I, human AAT was administered to OIR mouse pups by intraperitoneal (IP) injection in a 20 µl volume every second day between P7 and P12 (Fig. 1). To determine if AAT influenced retinal neovascularization and inflammation, separate cohorts of mice were administered human AAT as described above and in phase II OIR between P12 to P18 (Fig. 1). Litters of OIR mice were randomised to be treated with vehicle (controls) or AAT (120 mg/kg in vehicle comprised of formulated buffer; 144 mM mannitol, 38 mM sodium chloride and 17 mM sodium phosphate, pH 7.0, CSL Limited, Victoria, Australia). Comparisons were made to OIR mice administered human serum albumin, HSA, 120 mg/kg) by IP injection and room air controls. The dose and timing of AAT treatment is based on previous studies [19–21]. OIR mice were included in the study if they had consistent body weight gain in accordance with the established criteria for OIR studies [23]. As gender does not influence the development of OIR [26], both male and female mouse pups were studied. Mice were humanely killed at P12 or P18 with sodium pentobarbitone by IP injection (170 mg/ml, Virbac, Peakhurst, NSW, Australia).

### Histological analysis of microglial/macrophage density in retina

Microglia resident within the retina proliferate and become activated in response to tissue injury [27]. The density of microglia can be determined by their expression of ionised calcium binding adaptor protein-1 (Iba1), although this marker also identifies macrophages. Here, the density of microglia/macrophages in the retina was evaluated with immunohistochemistry as previously reported [28, 29]. Three µm paraffin serial sections were obtained from one retina from each animal. Four sections of retina at least 60 μm apart were randomly selected and incubated with 10% normal goat serum (5425 S, Cell Signalling Technology, MA, USA). Sections were then incubated overnight at 4 °C with a rabbit anti-Iba1 antibody (1:1000, 019-19741, Wako, Tokyo, Japan). Sections were washed with 0.1 M phosphate buffered saline pH 7.4 (PBS), incubated for 1 h with biotin-conjugated goat anti-rabbit IgG (1:200, E0432, DakoCytomation), washed with PBS and then incubated with the Vectastain ABC standard kit (Vector Laboratories, Newark, CA, USA) for 30 min and liquid DAB + substrate chromagen system (Dakocytomation) for 15 s. Sections were counterstained with Mayer’s Hematoxylin. For quantitation, 4 non-overlapping fields spanning the retina between the inner limiting membrane and inner plexiform layer, were captured at x400 magnification using a Nikon DS-Ri2 camera from each retina (Nikon Instruments Inc.). ImageJ software was used to set a threshold for immunolabeling which was applied to all fields. Data are presented as the percentage of Iba1 + cells per mm^2^. Investigators were blinded to the experimental mouse groups.

To verify the effect of AAT on Iba1 + cells, immunolabelling for Iba1 was performed on flatmounted retina. Flatmounts were incubated for 1 h at room temperature with 10% normal goat serum in 0.3% Triton-100 at room temperature, and then overnight at 4 °C with anti-mouse Iba1 (1:100, Wako) dissolved in 1% normal goat serum, 0.3% Triton X-100 and PBS. Six successive 10-minute washes were performed with wash buffer (0.3% Triton X-100 in PBS). Retinas were incubated for 1.5 h at room temperature with anti-rabbit IgG Alexa Fluor^®^ 548 (Molecular Probes, Eugene, OR, USA) dissolved in 1% normal goat serum and 0.3% Triton X-100. Retinas were washed 6 times and then incubated with FITC-conjugated isolectin (1: 100, L9381, Sigma) in 1% Triton X-100 to identify blood vessels. After washing, retinas were counterstained with DAPI (300 nM, Molecular Probes) to identify nuclei and mounted in fluorescent mounting medium (DakoCytomation). Retinal flatmounts were visualised with a Zeiss LSM800 scanning confocal microscope (Carl Zeiss AG, Oberkochen, Germany), with a 20x objective lens. Images were captured with Zen 2.3 software (Blue). Nine to twelve fields per retina were randomly selected from the ganglion cell layer to the inner plexiform layer. The density of Iba-1 + cells was quantitated by counting the number of Iba-1 + cells per field and presented as the number of Iba1 + cells per mm^2^.

### Quantitation of Iba1 + cell process length in retina

The activation of microglia can be visualised by the shortening of their cell processes [30]. Retinal flatmounts were prepared as described above and cell process length was measured from the centre of the cell soma to the process tip using Image J software (v3.1, National Institutes of Health, Bethesda, WA, USA) in cells located in the ganglion cell layer and inner plexiform layer. The primary process of each Iba1 + cell was measured as previously reported [23]. Data are presented as the cell process length (µm) of Iba1 + cells.

### Flow cytometry analysis for microglia and macrophages in retina

To further evaluate retinal microglia and macrophages, retinas were prepared for flow cytometry according to previous methods [11, 23]. Retinas were freshly dissected in Dulbecco’s phosphate buffer saline containing calcium and magnesium (D-PBS with Ca2^+^/Mg^+^, #14040182, Thermo Fisher Scientific) supplemented with 0.5% bovine serum albumin and immediately processed for enzymatic digestion with a commercially available kit (Neural Tissue Dissociation kit #130-094-802, Miltenyi Biotec, NSW, Australia) according to the manufacturer’s instructions for retinal tissues. Retinas were washed with 6 ml of D-PBS with Ca2^+^/Mg^+^ before transferring to a gentleMACS C tube (#130-096-334, Miltenyi Biotec) containing enzyme mixes from the kit for enzymatic digestion. Homogenization was performed using an automated dissociator (GentleMACS Octo Dissociator with Heaters, # 130-096-427, Miltenyi Biotec) according to the manufacturer’s instructions for neuronal tissues. Digested retinas were further homogenised with gentle trituration with a 1 ml pipette. After dissociation, retinal homogenates were washed with 6 ml of D-PBS with Ca2^+^/Mg^+^ and 0.5% BSA and filtered twice through a 70 mm strainer (BD Biosciences, San Jose, CA, USA). Cells were spun down at 300 g for 10 min at room temperature and suspended in 1 ml D-PBS with Ca2^+^/Mg^+^ supplemented with 0.5% bovine serum albumin for cell counting using an automated counter (Countess II FL, Thermo Fisher Scientific). One and a half million retinal cells were incubated in D-PBS without Ca2^+^/Mg^+^ (#14190250, Thermo Fisher Scientific) containing CD16/CD32 antibodies (Mouse BD Fc Block, #553142) to block non-specific binding and a dead cell stain (Aqua, #L34957, Thermo Fisher Scientific) to exclude dead cells. Cells were washed twice with FACS buffer and further incubated with an antibody cocktail consisting of CD45 BV786 (#564225), and CD11b AF700 (#557960) antibodies for 45 min at 4 °C. Flow cytometry was performed using a Fortessa X-20 (BD Biosciences) with at least 100,000 events. Cell viability was 95.84 ± 4.51% (*n* = 47 from 4 independent experiments). FlowJo software (v 10.8.1, Tree Star, Inc., OR, USA) was used for data analysis. Unless otherwise specified, all products were purchased from BD Biosciences.

### Quantitative PCR of retina

We utilised a previously published method [11, 23, 28], whereby total RNA was isolated from single retina using the RNeasy mini kit (Qiagen, Doncaster, VIC, Australia), and then 1 µg of RNA was subjected to DNase treatment (DNA-free kit, Ambion, Carlsbad, CA, USA) and reverse transcription (First Strand cDNA synthesis kit, Roche, Switzerland). mRNA expression was normalised to 18s rRNA endogenous control and the relative fold difference in expression was calculated using the comparative 2- ΔΔCt method. The primer sequences for *Vegfa* are forward primer: 5’-AGCAGAAGTCCCATGAAGTGATC-3 and reverse primer: 5’—TCAATCGGACGGC AGTAGCT-3’. The primer sequences for tumor necrosis factor (*Tnf*) are, forward primer: 5’-GCCTATGTCTCAGCCTCTTCTC-3’ and reverse primer: 5’-CACTTGGTGGTTTGCTACGA-3’. The primer sequences for monocyte chemoattract protein − 1 (*Mcp-1*) are, forward primer: 5’-CAGGTGTCCCAAAGAAGCTGTAG-3’ and reverse primer: 5’-GGGTCAGCACAGACCTCTCTCT-3’. The primer sequences for interleukin *Il*-*1b* are, forward primer: 5’-GTTCCCATTAGACAACTGCACTACA-3’ and reverse primer: 5’-CCGACAGCACGAGGCTTTT-3’. The primer sequences for *Il-6* are, forward primer: 5’-ACAAAGCCAGAGTCCTTCAGAGA-3’and reverse primer: 5’-CTTCTGTGACTCCAGCTTATCTGTTAG-3’.

### Analysis of vaso-obliteration and neovascularisation in retinal flatmounts

Retinal flatmounts were prepared as described previously [11, 23]. Eyes were enucleated and fixed in 4% paraformaldehyde in PBS for 30 min at room temperature. Retinal flatmounts were stained with fluorescein isothiocyanate-conjugated isolectin (FITC) GS-IB4 (L9381, Sigma, St Louis, MI, USA) or PBS only (negative control) and imaged using a Zeiss Axio (Carl Zeiss, Gottingen, Germany) microscope attached to a camera (AxioCam MRc, Carl Zeiss). Entire retinal montages were obtained using the tiling tool in the AxioObverser Software (v5.3, Carl Zeiss). ImageJ was used to quantitate vaso-obliteration and neovascularisation using the threshold tool. Mice from 2 to 3 different litters were evaluated at P12 and P18. Investigators were blinded to the experimental groups.

### Histological analysis of Müller cells in retina

Müller cells are macroglia that extend across almost the entire retina and are critical for maintenance of the blood-retinal barrier [31]. Müller cells obtain a gliotic phenotype in response to tissue stress and injury that can be visualised by their increased expression of glial fibrillary associated protein (GFAP) [31]. Three µm paraffin sections of retina were incubated overnight at 4 °C with a rabbit polyclonal anti-GFAP antibody (1:500, Z0334, DakoCytomation, Glostrup, Denmark). The sections were then washed with PBS and incubated for 1 h with Alexa Flour 488-conjugated goat anti-rabbit IgG (1:200, A-11008, Life Technologies, VIC, Australia). The sections were washed with PBS, counterstained with 4’,6-diamidino-2-3 phenylindole (DAPI, 0.5 µg/mL, D9542, Molecular Probes, Sigma) and coverslipped with Dako fluorescent mounting medium (S3023, DakoCytomation).

### Quantitation of GFAP immunolabelling in retina

The expression of GFAP was quantitated as previously reported [11, 23]. Four sections at least 60 μm apart were randomly selected from one eye from each animal. In each section, 4 non-overlapping fields from the central, mid and peripheral retina that span the entire retina were captured at x400 magnification using a Nikon DS-Ri2 camera (Nikon Instruments Inc. NY, USA). ImageJ software set a threshold for immunolabeling applied to all fields. Data are presented as the percentage of GFAP immunolabelling per field of central, mid, peripheral and whole retina. Investigators were masked to the experimental groups.

### ELISA

To further explore damage to the blood-retinal barrier, retinal vascular leakage was measured with an albumin ELISA, and VEGFA protein levels by ELISA in accordance with previous publications [11, 23]. To exclude any contribution of circulating protein from the measurements, mice were perfused via the heart with PBS (5 ml) prior to tissue collection. Retinas were digested in 200 µl of T-PER buffer (Invitrogen, Waltham, MA, USA) containing a phosphatase-protease inhibitor cocktail (1/100, Thermo Fisher Scientific, Victoria, Australia) using a Bullet Blender Tissue Homogenizer (Next Advance, NY, USA) for 5 min at speed 9 at 4 °C. Protein lysates were centrifuged at 10,000 rpm for 10 min at 4 °C and supernatants collected. Samples were run in duplicate for mouse albumin (#E-90AL, Immunology Consultants Laboratory, Portland, OR, USA) and mouse VEGFA (#DY493, R&D Systems, MN, USA) according to the manufacturer’s instructions. Total protein concentration of retinal homogenates was measured using a Bradford assay (Biorad). Albumin and VEGFA levels were normalised to the total protein concentration.

### Statistics

Data were first assessed for normality by Kolmogorov-Smirnov, D’Agostino’s and Pearson omnibus, as well as Shapiro-Wilk normality tests. For normally distributed data with equal variances, one-way ANOVA followed by Tukey’s post hoc test was used. For normally distributed data with unequal variances, Welch’s ANOVA followed by Dunnett’s T3 test was used. Non-parametric data were analysed using the Kruskal-Wallis test followed by Dunn’s post-test. The sample size was estimated by a power analysis assuming a normal distribution. P-values smaller than 0.05 were considered significant. Investigators were masked to the experimental groups. Values are expressed as mean ± SEM.

## Results

### Body weight is not influenced by AAT

As expected, OIR controls had reduced body weight at P12 and P18 compared to room air controls. AAT and the protein control, HSA, did not influence the body weight of room air controls or OIR controls (Table 1).

**Table 1:** Body weights of C57BL/6J mouse pups at P12 and P18. HSA, human serum albumin

Mouse groups	P12 Body weight (g)	*n*	P18 Body weight (g)	*n*
Room air control	7.2 ± 0.19	12	8.6 ± 0.14	40
Room air + HSA	NA		8.4 ± 0.15	19
OIR control	5.6 ± 0.06***	10	7.2 ± 0.06***	55
OIR + HSA	NA		7.4 ± 0.07***	25
OIR + AAT	5.9 ± 0.09**	13	7.5 ± 0.07***	61

AAT, alpha-1 antitrypsin. **p < 0.01, and ***p < 0.001 to room air controls. Values are mean ± SEM. *N* = 19 to 61 mice per group from 3 to 4 litters of mice per group. Data were analysed by Kruskal-Wallis test followed by Dunn’s test

### AAT levels are reduced in OIR

Reduced levels of AAT are associated with the development of some diseases [32, 33]. We found in phase I OIR at P12, circulating AAT levels in OIR mice (987.2 ± 28.32 µg/mL) were reduced compared to room air controls (1362 ± 32.68 µg/mL, *p* < 0.0001, *n* = 9 to 13 mice per group). At the end of phase II OIR at P18, circulating AAT levels in OIR mice (1512 ± 44.16 µg/mL) were like room air controls (1685 ± 76.92 µg/mL, *n* = 9 to 13 mice per group).

### AAT reduced Iba1 + cell density in phase II OIR

**Fig. 2:**
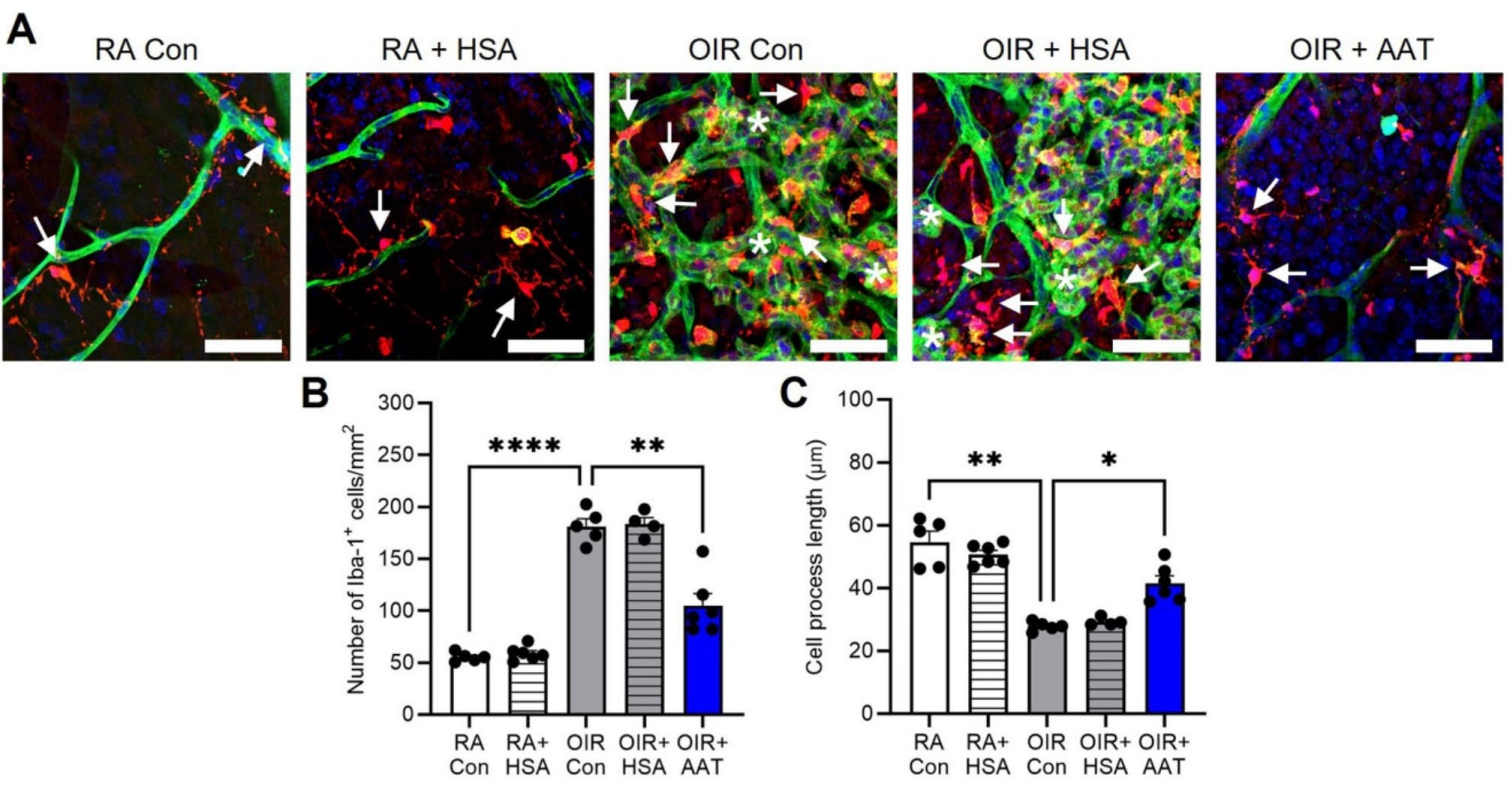
AAT reduced the number of Iba1 + cells and prevented the reduction in cell process length in phase II OIR mice at P18. RA, room air. Con, control. HSA, human serum albumin, AAT, alpha-1 antitrypsin. **(A)** Representative images of retinal flatmounts labelled with Iba1 showing microglia/macrophages (red, arrows), FITC-isolectin blood vessels (green), and DAPI nuclei (blue). Neovascular tufts are denoted by asterisks. Scale bar, 50 μm. **(B)** Quantitation of Iba1 + cells per mm^2^ within the ganglion cell layer and inner plexiform layer. **(C)** Quantitation of the primary cell process length of each Iba1^+^ cell within the ganglion cell layer and inner plexiform layer. *n* = 4 to 6 mice per group. Values are mean ± SEM. *p < 0.05, **p < 0.01, and ****p < 0.0001. Data were analysed by one-way ANOVA followed by Tukey’s test

At P18, few Iba1 + cells were identified in room air controls and room air + HSA (Supplemental Fig. 1). In OIR controls and OIR + HSA, the density of Iba1 + cells was similar and increased compared to room air mice (Supplemental Fig. 1). In OIR mice, treatment with AAT reduced the density of Iba1 + cells compared to OIR controls but not to the level of room air controls (Supplemental Fig. 1). Similar results were found in retinal flatmounts, with Iba1 + cells increased in OIR controls and OIR + HSA and often associated with neovascular tufts, compared to room air controls (Fig. 2A). In OIR mice treated with AAT, the number of Iba1 + cells in retinal flatmounts was reduced compared to OIR controls but not to the room air control levels (Fig. 2B).

### AAT restored cell process length of Iba1 + cells in phase II OIR

At P18, the cell process length of Iba1 + cells was similar between room air controls and room air + HSA (Fig. 2C). In OIR controls, Iba1 + cells had shorter cell processes compared to room air controls indicating an activated profile (Fig. 2C). In OIR mice treated with AAT, the cell processes of Iba1 + cells were longer compared to OIR controls and resembled room air controls (Fig. 2C).

### AAT reduced the number of macrophages in phase II OIR

**Fig. 3:**
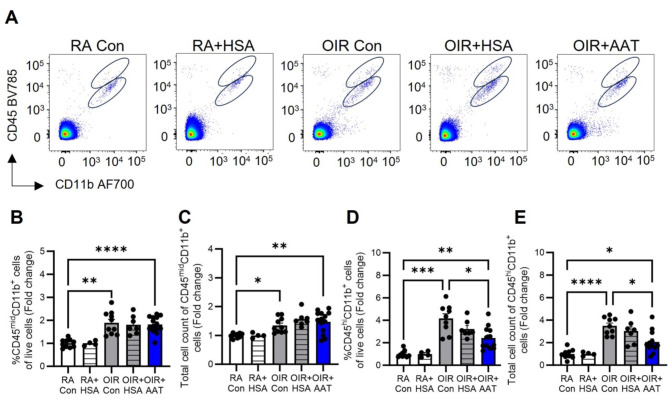
AAT reduced the number of macrophages in the retina of phase II OIR mice at postnatal day 18. RA, room air. Con, control. HSA, human serum albumin. AAT, alpha-1 antitrypsin. **A.** Gating strategy for CD45^mid^CD11b^+^ microglia and CD45^hi^CD11b^+^ macrophages. Fold change in the density (**B**) and total cell count (**C**) of CD45^mid^CD11b^+^ cells in retina. Fold change in the density (**D**) and total cell count (**E**) of CD45^hi^CD11b^+^ cells in retina. *n* = 4 to 16 mice per group. Values are mean ± SEM. *p < 0.05, **p < 0.01, ***p < 0.001, and ****p < 0.0001. Data were analysed by Welch’s ANOVA followed by Dunnett’s T3 test

As Iba1 immunolabelling does not distinguish between microglia and macrophages, flow cytometry of retina was performed. At P18, the density and total cell count of CD45^mid^CD11b^+^ microglia and CD45^hi^CD11b^+^ macrophages were increased to a similar extent in the retina of OIR controls and OIR + HSA compared to room air controls (Fig. 3A-E). Treatment of OIR mice with AAT reduced macrophages and not microglia in retina (Fig. 3A-E).

### Inflammatory factors in phase II OIR

**Fig. 4:**
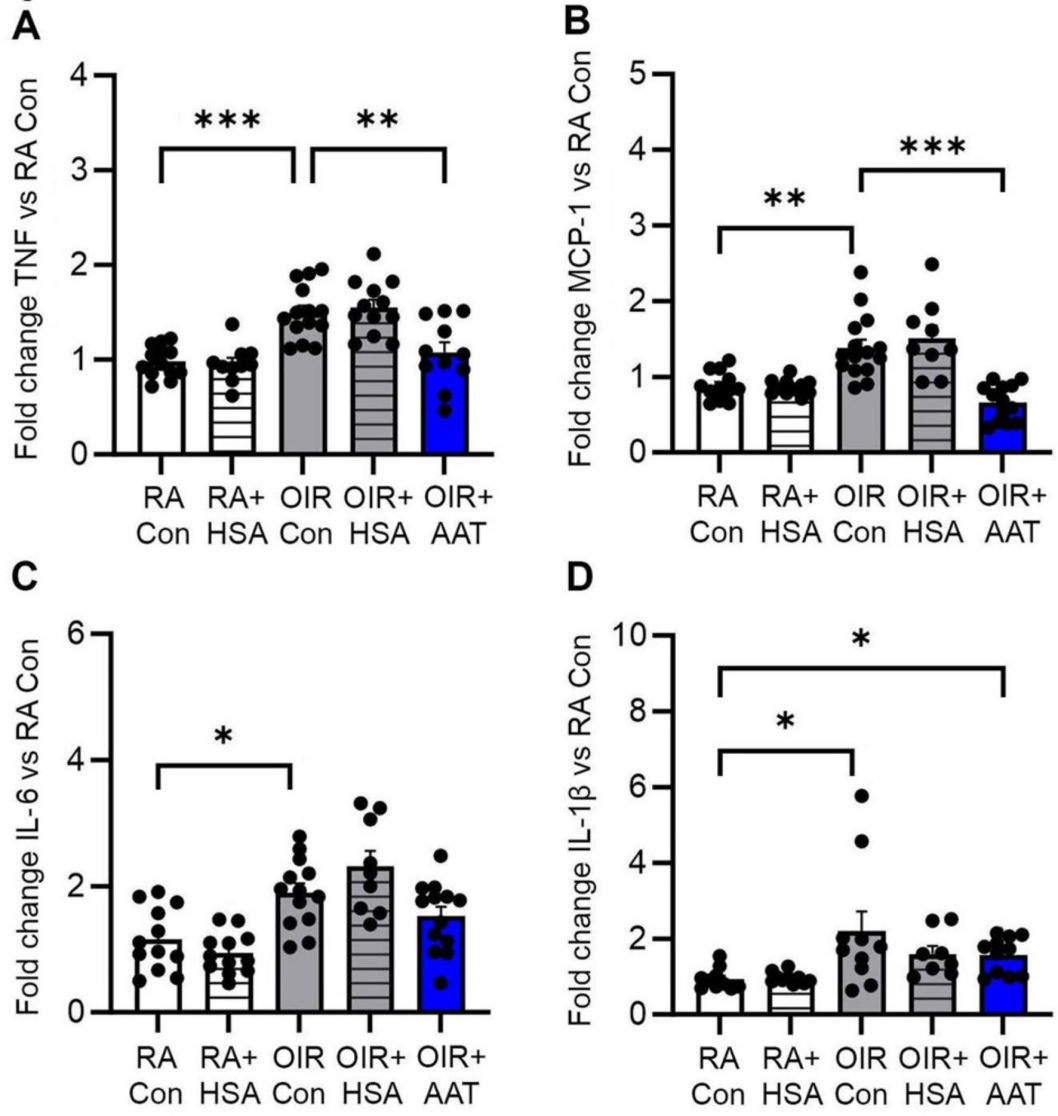
AAT reduced the expression of *Tnf* and *Mcp-1* in the retina of phase II OIR mice at P18. RA, room air. Con, control. HSA, human serum albumin. AAT, alpha-1 antitrypsin. mRNA levels of (**A)***Tnf*, (**B)***Mcp-1*, (**C)***Il-6*, and (**D**) *Il-1*b. *n* = 8 to 15 mice per group. Values are mean ± SEM. *p < 0.05, **p < 0.01, and ***p < 0.001. Data were analysed using one-way ANOVA followed by Tukey’s test or Welch’s ANOVA followed by Dunn’s test, or the Kruskal-Wallis test followed by Dunn’s test

Inflammation is associated with the neovascularization that develops in phase II OIR [34]. We therefore evaluated the expression of pro-inflammatory factors in the retina at P18. The mRNA expression of *Tnf*, *Mcp-1*, *Il-6*, and *Il-1b* were increased in the retina of OIR controls compared to room air controls (Fig. 4A-D). Treatment of OIR mice with AAT reduced the mRNA levels of *Tnf*, and *Mcp-1*, but not *Il-6* and *Il-1b* compared to OIR controls (Fig. 4A-D). In the OIR + AAT group, the mRNA levels of *Il-1b* remained increased compared to room air controls (Fig. 4D).

### AAT reduced retinal vaso-obliteration and neovascularisation

**Fig. 5:**
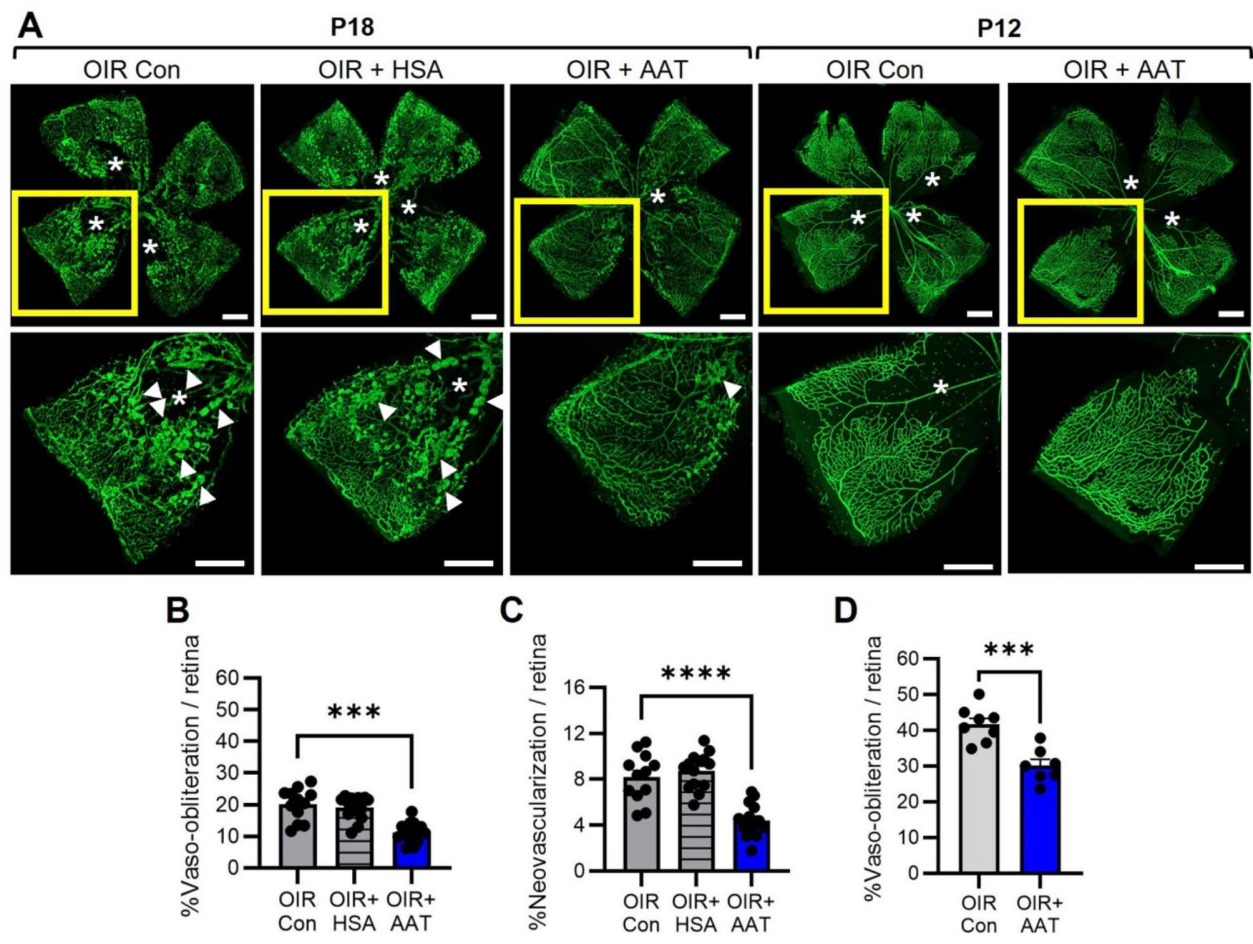
AAT reduced vascular pathology in retina of OIR mice at P18 and P12. Con, control. HSA, human serum albumin. AAT, alpha-1 antitrypsin. **A**. Representative images of retinal flatmounts stained with FITC-isolectin to show blood vessels (green). Top panels show whole retina. A quadrant of retina (yellow box) is magnified in the lower panel. Asterisks denote vaso-obliteration. Arrowheads denote neovascularisation. Scale bar, 500 μm. **B**. Quantitation of vaso-obliteration at P18. **C.** Quantitation of neovascularisation at P18. **D**. Quantitation of vaso-obliteration at P12. *n* = 7 to 15 mice from 2 to 3 litters of mice per group. ***p < 0.001, and ****p < 0.0001. Data were analysed using one-way ANOVA followed by Tukey’s test or Welch’s ANOVA followed by Dunnett’s T3 test, or unpaired *t* test. Values are mean ± SEM

In phase I OIR at P18, AAT reduced neovascularisation and vaso-obliteration in retina by almost 50% compared to OIR controls and OIR + HSA (Fig. 5A-C). We next evaluated phase I OIR when retinal vaso-obliteration is maximal. In OIR control mice, retinal vaso-obliteration at P12 was approximately twice as severe than at P18 (Fig. 5B, D). In OIR mice at P12, treatment with AAT treatment reduced retinal vaso-obliteration compared to OIR controls (Fig. 5D).

### AAT reduced retinal gliosis, VEGFA and vascular leakage in phase II OIR

**Fig. 6:**
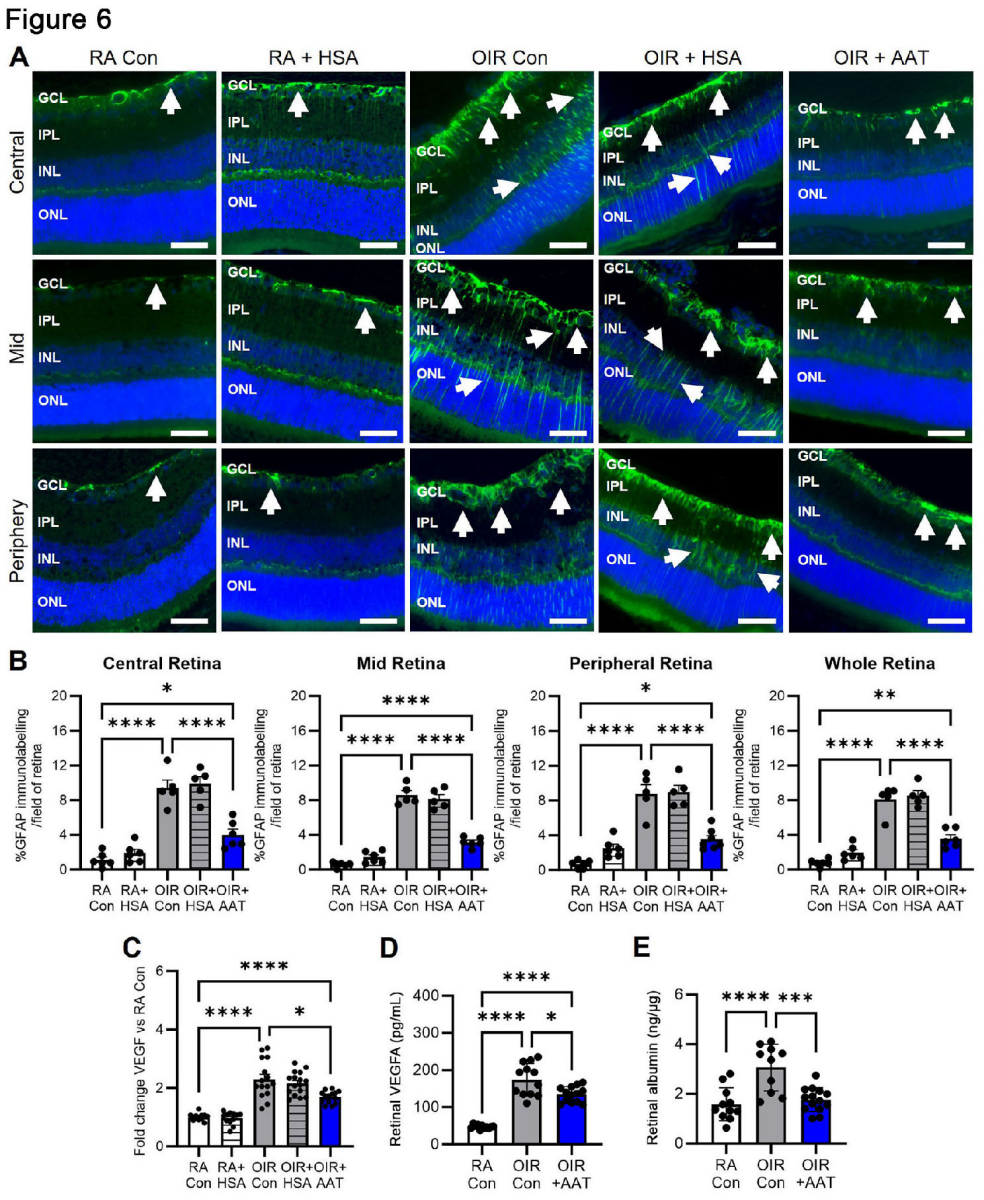
AAT reduced retinal gliosis, VEGFA, and vascular leakage in phase II OIR mice at P18. RA, room air. Con, control. HSA, human serum albumin. AAT, alpha-1 antitrypsin. **(A)** Representative 3 μm paraffin sections of retina showing GFAP immunolabelling (green) in Müller cell processes (arrows). Nuclei counterstained with DAPI (blue). GCL, ganglion cell layer. IPL, inner plexiform layer. INL, inner nuclear layer. ONL, outer nuclear layer. Scale bar, 50 μm. **(B)** Quantitation of GFAP in the central, mid, peripheral and whole retina by One-way ANOVA. *n* = 5 to 6 mice per group. *p < 0.05, **p < 0.01, and ****p < 0.0001. **C.***Vegfa* mRNA in retina. **D.** VEGFA protein in retina from mice perfused with PBS. **E.** Retinal vascular leakage (albumin ELISA) in retina from mice perfused with PBS. Values are mean ± SEM. *n* = 9 to 15 mice per group. *p < 0.05, ***p < 0.001, and ****p < 0.0001. Data were analysed by Welch ANOVA followed by Dunnett’s T3 tests and one-way ANOVA followed by Tukey’s test

GFAP immunolabelling in room air controls and room air + HSA at P18 was similar and confined to the surface of the retina in the region of the inner limiting membrane and ganglion cell layer in the mid, central and peripheral retina (Fig. 6A, B). In OIR controls and OIR + HSA, GFAP immunolabelling was increased to a similar extent compared to room air control mice, extending throughout the retina in Müller cell processes (Fig. 6A, B). In OIR mice, treatment with AAT reduced GFAP immunolabelling in all regions of the retina compared to OIR controls (Fig. 6A, B).

VEGFA can stimulate vascular leakage in the retina [35]. In OIR controls at P18, *Vegfa* mRNA levels in retina were increased compared to room air controls (Fig. 6C). In OIR mice, treatment with AAT reduced *Vegfa* mRNA levels compared to OIR controls (Fig. 6C). VEGFA protein levels and vascular leakage in retina were increased in OIR controls compared to room air controls. In OIR mice both VEGFA protein and vascular leakage in retina were reduced by AAT treatment (Fig. 6D, E).

## Discussion

The major findings of this study are that circulating AAT levels are reduced in OIR, and AAT treatment attenuates key aspects of retinal vasculopathy including vaso-obliteration, neovascularisation, gliosis, VEGFA, and vascular leakage. It is yet to be fully understood if AAT directly influences the retinal vasculature and/or protects the vasculature through its anti-gliotic and anti-inflammatory properties as evidenced by reductions in *Tnf* and *Mcp-1* mRNA levels. The source of these inflammatory factors was not investigated, but microglia/macrophages are a possibility with AAT reducing their density in retina.

Inflammation is a major contributor to retinal vascular disease and is mediated by certain cytokines [36–39]. There is considerable evidence that AAT reduces pro-inflammatory molecules such as TNF, MCP-1, IL-6, and IL-1ß [40, 41]. In the current study, AAT reduced the mRNA expression of *Tnf* and *Mcp-1* in the retina in phase II OIR at P18. TNF is a well-known causal factor in the development of retinal vascular disease [11, 36] and therefore AAT’s reduction of TNF levels in OIR is noteworthy. With respect to MCP-1, AAT’s influence is likely multifaceted, with evidence suggesting that serine proteases like cathepsin G indirectly increase MCP-1 expression and activity through protease-activated receptors and inflammatory mediators [42]. Relevant to OIR are data demonstrating that MCP-1 blocking antibodies reduced retinal neovascularisation [34]. The reason for the inability of AAT to reduce IL-6 and IL-1ß at P18 in OIR is unclear but may relate to the phase of OIR with reports that IL-1ß expression in microglia is particularly elevated in phase I OIR [43].

In response to the tissue stress and relative hypoxia that occurs in phase II OIR, microglia undergo distinct cellular changes that result in a pro-inflammatory phenotype [10, 37, 44, 45]. This includes cell proliferation in the inner retina and associated with the vasculature, and cell activation involving their transition from a ramified to ameboid appearance with shorter cell processes and the expression of activation markers [23]. Our morphological analysis of Iba1 + cells revealed that AAT reduced the number of microglia/macrophage cells and partially prevented the phenotypic changes associated with microglial activation, consistent with previous reports in models of ocular hypertension and retinal degeneration showing AAT reducing Iba1 + cells [21, 22]. However, our flow cytometry data revealed a more nuanced response. AAT treatment reduced the number of retinal macrophages (CD45^hi^CD11b+), but not microglia (CD45^mid^CD11b+). This reduction in macrophages suggests that these cells participate in mediating the protective effects of AAT, as they have been shown to promote inflammation and neovascularisation in OIR [46]. An outstanding question is whether the retinal microglia/macrophage population are a source of AAT with previous studies identifying that some microglia/macrophages (positive for Iba1) are immunolabelled for AAT, and AAT expression is reduced in retinal neurodegeneration [22]. Our finding that plasma levels of AAT are reduced in phase I OIR suggests that retinal AAT may in part be derived from the circulation.

Neovascularisation in the inner retina involves the growth of abnormally formed vessels into the vitreous cavity. It is a hallmark feature of retinopathy of prematurity and diabetic retinopathy and is accompanied by the leakage of fluid and protein into the retina and vitreous cavity from a compromised blood-retinal barrier [5, 47]. The OIR model recapitulates many of the features of retinopathy of prematurity but is also used to study the retinal vasculopathy that develops in proliferative diabetic retinopathy, albeit the hyperglycaemia of diabetes is absent [48]. In the current study, we demonstrated that AAT administered at the commencement of phase II OIR, reduced retinal neovascularisation, a finding that to our knowledge has not been previously reported. There is some evidence from other disease models, that AAT influences blood vessel growth such as inhibiting tumour angiogenesis, neovascularisation in the rat cornea and the chemotaxis of human microvascular endothelial cells [49, 50]. Retinal neovascularisation in OIR is influenced by the extent of vaso-obliteration that peaks in phase I as a response to high oxygen exposure that disrupts physiological angiogenesis [47]. AAT’s reduction of vaso-obliteration in phase I OIR is therefore important, however the precise mechanisms of action are currently unclear, but could relate to the ability of AAT to suppress endothelial cell apoptosis [51]. Overall, our findings build on previous research in the retina identifying that AAT attenuates neurodegeneration by preventing ganglion cell loss in diabetic animals [20], promotes the survival of transplanted inducible pluripotent stem cells in mice with ocular hypertension [21], and reduces retinal photoreceptor dysfunction in a mouse model of retinitis pigmentosa [22].

Macroglial Müller cells have an important role in the development of retinal vasculopathy due to their close association with the vasculature [31]. In OIR, Müller cells undergo reactive gliosis in response to the relative tissue hypoxia caused by mice transitioning from a high oxygen environment in phase I OIR to room air in phase II OIR. Accompanying this gliotic phenotype is the increased production of VEGFA by Müller cells which stimulates retinal neovascularisation and vascular permeability [3, 4]. In the current study, AAT treatment during phase II OIR reduced Müller cell gliosis in the central, mid and peripheral regions, and elevated VEGFA mRNA and protein levels in the retina. If AAT directly influences VEGFA expression and secretion is not fully understood. However, it has been suggested that in diabetic retinopathy certain metalloproteinases involved in regulation of the extracellular matrix and vascular basement membrane thickening are inhibited by AAT, resulting in reduced VEGFA levels and potentially vasculopathy [52, 53]. In terms of our finding that AAT reduced vascular permeability in the OIR retina, these results are consistent with reports that AAT protected the immature mouse brain from hypoxic injury including breakdown of the blood-brain barrier [54].

## Conclusions

The findings of this study indicate that AAT can protect the retinal vasculature against vision threatening disease. Our finding that AAT levels are reduced in the circulation of mice in phase I OIR are in agreement with studies in patients with diabetic retinopathy showing reduced AAT levels [32, 33] and activity [55]. Evidence that AAT augmentation is beneficial in diabetic models [56, 57] and in our study of OIR suggests that AAT is a potential treatment approach for these neovascular retinopathies.

## Electronic supplementary material

Below is the link to the electronic supplementary material.

Supplementary Material 1

## Data Availability

The datasets used and/or analysed during the current study are available from the corresponding author on reasonable request.

## Abbreviations

Alpha-1: Antitrypsin AAT
DAPI: 4’,6-diamidino-2-3 phenylindole
FITC: Fluorescein isothiocyanate
GFAP: Glial fibrillary acidic protein
HSA: Human serum albumin
Iba1: Ionised binding adaptor protein 1
IL: Interleukin
IP: Intraperitoneal
MCP-1: Monocyte chemoattractant protein 1
MFI: Mean fluorescent intensity
OIR: Oxygen-induced retinopathy
P: Postnatal day
PBS: Phosphate buffered saline
PCR: Polymerase chain reaction
TNF: Tumour necrosis factor
VEGFA: Vascular endothelial growth factorA
