# Supplementary material for: Alpha-1 antitrypsin reduces inflammation and vasculopathy in mice with oxygen-induced retinopathy
Source: J Inflamm (Lond). 2025 Feb 11;22:6. doi: 10.1186/s12950-025-00431-3 (PMC11817893; doi:10.1186/s12950-025-00431-3)
Supplement: Supplementary file 1 — Supplementary Material 1 [file 12950_2025_431_MOESM1_ESM.docx]

**SUPPLEMENTAL MATERIAL**

**ALPHA-1 ANTITRYPSIN REDUCES INFLAMMATION AND VASCULOPATHY IN MICE WITH OXYGEN-INDUCED RETINOPATHY**

Varaporn Suphapimol^1^, Yu-Han Liu^2^, Sandro Prato^2^, Alexander Karnowski^2^, Charles Hardy^2^, Adriana Baz Morelli^2^, Abhirup Jayasimhan^1^, Devy Deliyanti^1^, and Jennifer L. Wilkinson-Berka^1^

^1^Department of Anatomy and Physiology, School of Biomedical Sciences, The University of Melbourne, Parkville, Victoria, Australia

^2^CSL Limited, Melbourne, Victoria, Australia

**Address for Correspondence:**

Professor Jennifer L. Wilkinson-Berka

School of Biomedical Sciences, The University of Melbourne

Level 2, Medical Building 181, Grattan Street, Parkville, Victoria, Australia, 3010

Email: [jennifer.wilkinsonberka@unimelb.edu.au](mailto:jennifer.wilkinsonberka@unimelb.edu.au)

**
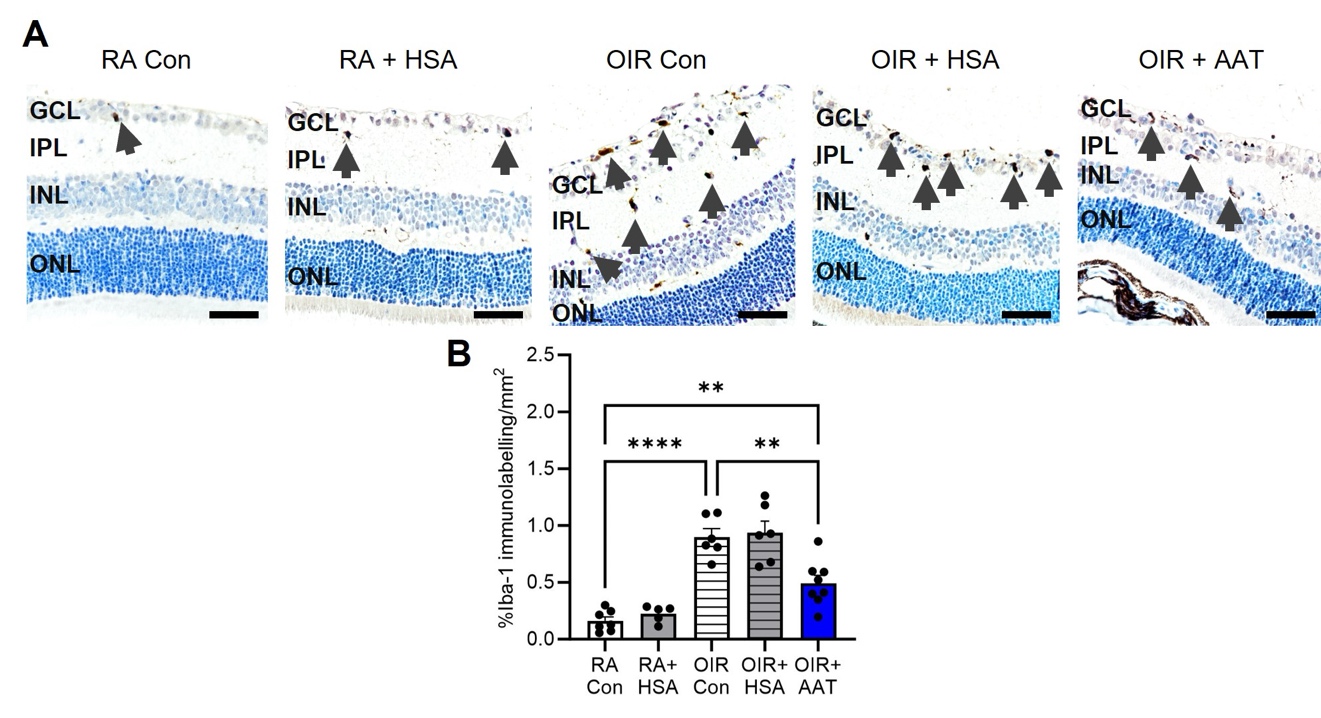
**

**Supplemental Fig. 1** Treatment with AAT reduced the density of Iba1+ cells (microglia/macrophages) in the retina of OIR mice at P18. RA, room air. Con, control. HSA, human serum albumin, AAT, alpha-1 antitrypsin. **A.** Representative images of Iba1+ cells (arrows) in 3 μm paraffin sections of retina counterstained with haematoxylin. GCL, ganglion cell layer. IPL, inner plexiform layer. INL, inner nuclear layer. ONL, outer nuclear layer. **B.** Quantitation of the percentage of Iba1 immunolabelling per mm^2^ within the region of the inner limiting membrane and inner plexiform layer. *n* = 5 to 8 mice per group. Scale bar, 50 µm. Values are mean ± SEM. ***p* < 0.01, and *****p* < 0.0001. Data were analysed by one-way ANOVA followed by Tukey's test.
